# Hypoxia in cervical cancer: from biology to imaging

**DOI:** 10.1007/s40336-017-0238-7

**Published:** 2017-07-10

**Authors:** Heidi Lyng, Eirik Malinen

**Affiliations:** 1 Department of Radiation Biology, Institute for Cancer Research, Norwegian Radium Hospital, Oslo University Hospital, Oslo, Norway; 2 Department of Medical Physics, Norwegian Radium Hospital, Oslo University Hospital, Oslo, Norway; 3 Department of Physics, University of Oslo, Oslo, Norway

**Keywords:** Cervical cancer, Imaging, Hypoxia, Positron emission tomography, Magnetic resonance imaging, Treatment outcome

## Abstract

**Purpose:**

Hypoxia imaging may improve identification of cervical cancer patients at risk of treatment failure and be utilized in treatment planning and monitoring, but its clinical potential is far from fully realized. Here, we briefly describe the biology of hypoxia in cervix tumors of relevance for imaging, and evaluate positron emission tomography (PET) and magnetic resonance imaging (MRI) techniques that have shown promise for assessing hypoxia in a clinical setting. We further discuss emerging imaging approaches, and how imaging can play a role in future treatment strategies to target hypoxia.

**Methods:**

We performed a PubMed literature search, using keywords related to imaging and hypoxia in cervical cancer, with a particular emphasis on studies correlating imaging with other hypoxia measures and treatment outcome.

**Results:**

Only a few and rather small studies have utilized PET with tracers specific for hypoxia, and no firm conclusions regarding preferred tracer or clinical potential can be drawn so far. Most studies address indirect hypoxia imaging with dynamic contrast-enhanced techniques. Strong evidences for a role of these techniques in hypoxia imaging have been presented. Pre-treatment images have shown significant association to outcome in several studies, and images acquired during fractionated radiotherapy may further improve risk stratification. Multiparametric MRI and multimodality PET/MRI enable combined imaging of factors of relevance for tumor hypoxia and warrant further investigation.

**Conclusions:**

Several imaging approaches have shown promise for hypoxia imaging in cervical cancer. Evaluation in large clinical trials is required to decide upon the optimal modality and approach.

## Introduction

Tumor hypoxia is an adverse factor in cervical cancer and associated with poor outcome regardless of treatment modality [1–4]. Therapeutic strategies to handle hypoxia are, therefore, highly needed, especially for locally advanced stages, where radiotherapy, often combined with cisplatin, is the main treatment option. Although local recurrence occurs in only about 10% of the cases, more than 30% experience distant relapse [5]. For stage III–IVa disease, the numbers are even higher. During radiotherapy, high radiation doses are applied to increase the likelihood of tumor control. Thus, many patients face severe damage to critical organs in the pelvis after therapy, including fistulas and rectal bleeding [5]. Strategies for modifying tumor hypoxia by normobaric or hyperbaric oxygen, or by use of nitroimidazole compounds as radiosensitizer, have been shown to improve radiotherapy outcome [6]. Modern approaches, combining radiation with molecular targeted drugs or escalating the radiation dose to hypoxic tumor regions, are on the agenda [7, 8], but such approaches strongly rely on robust hypoxia biomarkers. Medical imaging could allow for visualization of hypoxia within the entire tumor prior to and during therapy. In this respect, imaging is superior to the traditional invasive biomarker assays based on needle electrodes or tissue sampling.

Imaging is the cornerstone in diagnosis, radiotherapy planning, and treatment monitoring of cervical cancer [9]. Multimodality imaging with T2-weighted magnetic resonance imaging (MRI) and computed tomography (CT) is current state of the art for defining target volumes and calculating radiation doses delivered to the patient both for the external radiation therapy and for the brachytherapy. Fluorine 18-fluorodeoxyglucose (18F-FDG) positron emission tomography/computed tomography (PET/CT) may aid detection of pathological lymph nodes, and is becoming increasingly common for defining nodal targets. Functional MRI, including dynamic contrast-enhanced (DCE) and diffusion-weighted (DW) MRI, may improve target definition and is part of the standard imaging protocol at many institutions. In addition, recent technological developments, such as the PET/MRI hybrid scanners and MRI linear accelerator, will facilitate more advanced use of imaging in years to come. The first experience with PET/MRI in cervical cancer has shown improved integration of anatomical and functional tumor characteristics in MR and PET images and more reliable target definition as compared with PET/CT [10].

To date, imaging has almost exclusively been used for assessing anatomical features, such as tumor size, stage, and spread of the disease. There are emerging data showing that PET and MRI have a huge potential in visualization of hypoxia in cervix tumors. Here, we review current biological knowledge of hypoxia and hypoxia markers of relevance for imaging. We further discuss imaging techniques that have shown promise in a clinical setting to evaluate hypoxia and predict and monitor treatment outcome in cervical cancer, and address how these may play a role in new strategies to improve treatment outcome.

## The biology of hypoxia

### Tumor oxygen levels

Earlier studies assessing oxygen tension (*p*O_2_) in tumors by use of the Eppendorf electrodes have clearly demonstrated that hypoxia is a common feature of cervical cancer. Cohort-based median *p*O_2_ levels ranging from 2 mmHg (0.3% O_2_) to 14 mmHg (1.8% O_2_), as measured before the start of treatment, have been reported [1, 3, 11–13]. There are, however, considerable differences between individual tumors, and hypoxic fraction, in terms of *p*O_2_ readings below 5 mmHg (0.7% O_2_), can differ from 0 and up to 100%. A large pre-treatment hypoxic volume has been associated with the presence of lymph node metastases at diagnosis and poor overall or disease free survival [1–4]. Similar associations have been reported for locoregional control, but the data are less consistent probably due to few relapses in small patient cohorts. The significance is generally retained in multivariate analysis with clinical markers such as tumor stage and size [1, 2, 4]. Assessment of hypoxia status would, therefore, add valuable information to the traditional diagnostics in treatment planning.

Although some tumors are more hypoxic than others, a large intratumor heterogeneity in the oxygen levels exists, and sampling from several regions is required to achieve reliable hypoxia estimates that can be compared across patients [14]. Imaging is particularly appealing in this respect by providing information from the entire tumor. A challenge is, however, the small size of the hypoxic regions, which can be below the spatial resolution of medical images. Hence, *p*O_2_ levels ranging from 1 to 20 mmHg (0.1–2.6% O_2_) over a distance of less than 1 mm have been measured [15], and even smaller hypoxic patches of a couple of cell diameters have been detected with immunohistochemistry, using the hypoxia marker pimonidazole [16].

Tumor hypoxia is not static, but evolves and diminishes in a dynamic process depending on tumor growth, neo-angiogenesis, and treatment [17]. Thus, temporal fluctuation in *p*O_2_ over short time periods of less than 1 h has been detected in cervical cancer xenografts [18]. In an orthotopic cervical cancer mouse model, exposing the mice to varying O_2_ concentrations in cycles during tumor growth was shown to enhance the capability of tumor cells to metastasize to local lymph nodes [19]. This suggests that O_2_ fluctuations could be of clinical relevance. There is a lack of information on cycling hypoxia in human tumors, and imaging could facilitate such investigations by repetitive non-invasive measurements.

Changes in tumor hypoxia have been measured during fractionated radiotherapy [13, 20–22]. In a study comparing *p*O_2_ data before treatment and after 10 Gy of radiation, increased oxygenation was primarily found in the most oxygenated tumors, while the hypoxic tumors showed no change or a decrease [22]. This observation was attributed to more treatment-induced cell death and thereby probably a larger decrease in oxygen consumption in oxygenated tumors. The benefit of assessing hypoxia during therapy for outcome prediction is, however, not clear. Suzuki et al. [13] found a stronger association with locoregional control for *p*O_2_ data measured after 2 weeks of external radiation, whereas pre-treatment data performed better regardless of end-point in the study by Lyng et al. [4]. Tumor *p*O_2_ measured after 26–52 Gy of external radiotherapy seems to be less useful [21].

### Physiological and molecular hypoxia markers

Hypoxic tumors show specific physiological and molecular characteristics that can be visualized in medical images. Hypoxia occurs in poorly vascularized tumors, where the impaired oxygen supply is not capable of meeting the oxygen demand of the cells. Necrosis may develop under severe, long-lasting hypoxia, depending on the cells’ ability to adapt to the oxygen and nutrient deprived conditions. Hence, a negative correlation has been found between fraction of necrosis and *p*O_2_ in cervix tumors [23]. Several studies have addressed relationships between hypoxia and immunohistochemistry markers of oxygen supply and demand. Poorly vascularized tumors or tumor regions have shown low *p*O_2_ [24, 25], and long intercapillary distance has been associated with locoregional relapse [25]. Vascular parameters may, therefore, serve as surrogate markers of hypoxia in cervix tumors before the start of treatment. However, associations with poor outcome have also been found in cases of high vascular density in hot spots, which may reflect high angiogenic activity not necessarily related to hypoxia [25, 26]. Treatment-induced cell death has been shown to be more important than vascular changes for reoxygenation during the early phase of radiotherapy, probably because only small changes in vascular density have occurred at this stage [22]. Tumor cellularity, therefore, seems to also influence the hypoxia status, most likely reflecting the oxygen demand.

Tumor cells adapt to the hypoxic environment partly through stabilization of hypoxia inducible factors HIF1A and EPAS1 (HIF2A), enhancing glycolytic activity to maintain ATP levels when mitochondrial activity has slowed down [27]. Proteins involved in glucose metabolism have been investigated as possible endogenous hypoxia markers in immunohistochemical studies. High expression of HIF1A and its target proteins glucose transporter SLC2A1 (GLUT1) and pH regulator CA9 has been found in cervix tumors that were identified as hypoxic by pimonidazole staining or electrode measurements [28–32]. However, the spatial overlap between protein expression and pimonidazole staining has been found to be poor in many tumors [28, 31]. Moreover, although significant association between clinical outcome and expression of HIF1A, SLC2A1, CA9, or the glycolytic enzymes HK2 and PFKM2 has been found both for early [33–35] and late stage disease [32, 36–43], conflicting results have been reported [29, 30, 41]. This can probably be explained, because HIF1A and its target genes may be regulated by other factors than hypoxia, including reactive oxygen species (ROS), oncogenes, and metabolic stressors like lactate [27]. In addition, tumor cells rely in general on glycolysis rather than oxidative phosphorylation as energy source even in the presence of oxygen, a phenomenon termed the Warburg effect [27]. Rapidly proliferating cells can, therefore, have high glycolytic activity and express proteins involved in glucose metabolism regardless of hypoxia.

In addition to activation of the HIF pathway, hypoxia tolerance in tumors is also mediated by activation of the unfolded protein response (UPR) and inhibition of MTOR signaling [44]. In global gene expression studies, we found that both HIF targets and UPR genes were required to construct a robust hypoxia gene classifier that could be validated across two independent cervical cancer cohorts [45, 46]. The importance of UPR has been further emphasized by the observations that high expression of the UPR regulated protein LAMP3 promoted hypoxia-driven metastasis in an orthotopic cervical cancer model and was associated with hypoxia in patient tumors [47]. Increased understanding of the mechanisms underlying hypoxia-related aggressiveness will be important for development of new molecular imaging approaches.

## Positron emission tomography (PET)

PET employing tracers specific for low oxygen concentrations in tissue is the most direct method for non-invasive 3D imaging of hypoxia [48–50]. In PET, tracers that carry a positron emitting radioisotope such as 18-Fluorine (18F) or 11-Carbon (11C) are intravenously administered. Following radioactive decay in the body, the positron may typically travel up to 1 mm before annihilating with an atomic electron. Subsequently, two high-energy photons are created in opposite directions at approximately 180 degrees from each other. The photon pair may be registered in the PET ring detector system as a so-called co-incidence, connecting a line of response through the patient and thus providing the basis for image formation. After acquiring sufficient number of co-incidences, which typically takes some minutes, a 3D image series are reconstructed most often alongside the CT image series provided by the PET/CT scanner.

### Hypoxia PET tracers and uptake mechanisms

There are many properties that characterize a PET tracer’s specificity to tumor hypoxia [48]. Of these are (1) retention in hypoxic but not normoxic tissue, (2) kinetics suitable for imaging, (3) appropriate lipo- and hydrophilicity for optimal cell membrane transport and clearance of non-bound tracer, and (4) reflection of the return to normoxic conditions, if relevant. There are mainly two groups of hypoxia PET tracers: fluorine labeled nitroimidazoles and copper labeled diacetyl-*bis*(*N*4-methylthiosemicarbazone) (ATSM) analogues. Nitroimidazoles accumulate in cells by first a passive diffusion across the cell membranes. Thereafter, if hypoxia is present, the compound’s nitro group undergoes chemical reduction in a multi-stage process mediated by nitroreductase enzymes. The intermediate products in this process are highly reactive and can bind to macromolecules in the cell, thereby fixating the reduced form of the tracer intracellularly. If oxygen is present, the reductive process is reversed and no reactive intermediates are formed [51]. Moreover, nitroimidazoles will not accumulate in necrotic tissues as active uptake is dependent on functioning nitroreductase enzymes [52].

Cu-ATSM belongs to the group of dithiosemicarbazones, which are chemically quite different, and thus show other pharmacokinetic properties compared to nitroimidazoles. Several positron emitting copper isotopes such as 60Cu and 64Cu may be used for complexing with ATSM [53]. Compared to 18F, e.g., 64Cu has much longer half-life, which may facilitate wider distribution to centers not having PET cyclotron. In addition, the emitted positrons have shorter range, which may result in images with slightly higher resolution. However, Cu-ATSM uptake in hypoxic tissues is not completely understood [54]. The tracer has high membrane permeability, thereby allowing efficient diffusion. Intracellularly, it is believed that the Cu^2+^ part of the complex is irreversibly reduced to Cu^+^ under hypoxia, possibly leading to the dissociation of Cu^+^-ATSM and trapping of the Cu^+^-ion. In the presence of oxygen, the reduction of Cu^2+^ may be counteracted by reoxidation, thus preventing dissociation of the complex and resulting in clearance from normoxic tissues [55].

### Nitroimidazoles as hypoxia tracer

The most widespread nitroimidazole compound for hypoxia imaging is 18F-fluoromisonidazole (18F-FMISO) [52]. The tracer is highly lipophilic, ensuring high diffusion across cell membranes. However, this also causes very slow clearance of the tracer from normoxic tissue, making the tumor to blood ratio rather low. For optimal discrimination between hypoxic and normoxic tissue with 18F-FMISO PET, long intervals of typically 2 h between tracer injection and imaging are required [56]. Although the tracer has been extensively studied in a variety of cancers both pre-clinically and clinically, very little has been published on cervical cancer. In a recent study on 11 patients, pre-treatment 18F-FMISO PET indicated the presence of hypoxia in all tumors, where the extent of hypoxia was independent of tumor volume (Table 1) [57]. Neither associations between 18F-FMISO uptake and outcome nor relations to immunohistopathological or genetic markers were reported. The patients also underwent PET with 18F-FDG and multiparametric DCE- and DW-MRI, and the strongest correlation was found between maximum tumor uptake of 18F-FMISO and 18F-FDG.

**Table 1:** Selected hypoxia imaging studies in primary cervical cancer

Modality	Image parameter	References	No. of pts	Biological end-point	Clinical end-point
PET					
18F-FMISO	Uptake	[57]	11	18F-FDG PET (+)	Feasibility
18F-FAZA	Visual grading	[59]	15	ND	Survival (NS)
18F-FETNIM	*T*/*M*	[60]	16	Serum osteopontin (NS)	Survival (÷)
				18F-FDG PET (NS)	
60Cu-ATSM	*T*/*M*	[64]	15	CA9 expression (+)	Survival (÷)
		[62]	38	18F-FDG PET (NS)	Survival (÷)
MRI					
BOLD	Mean signal	[80]	30	ND	Tumor shrinkage (÷)
		[82]	65	ND	Short term treatment response (÷)
DCE-MRI	RSI	[86]	50	*p*O_2_ (+)	ND
		[87]	12 + 8	*p*O_2_ (+); cell density (÷)	ND
		[89]	37	Vessel density (+)	ND
		[96]	81	ND	Locoregional control (+); survival (+)
		[98]	13	ND	Tumor regression (+)
		[101]	98	ND	Locoregional control (+)
					Survival (+)
DCE-MRI	EF	[97]	85	ND	Survival (+)
DCE-MRI	K^trans^	[98]	13	ND	Tumor regression (+)
		[92]	78	ND	Locoregional control (+); survival (+)
DCE-MRI	A_Brix_	[86]	50	*p*O_2_ (+)	Survival (+)
		[99]	57	Vessel density (+)	Survival (NS)
		[46]	78	Gene expression (÷, 46 pts)	Survival (+)
CT					
DCE-CT	BF	[88]	32	*p*O_2_ (+)	ND
DCE-CT	BV	[100]	93	ND	Tumor response (+)

Pts, patients; T/M, tumor to muscle ratio; RSI, relative signal increase; EF, enhancing fraction; BF, tumor blood flow; BV, blood volume; +, significant positive association with image parameter; ÷, significant negative association with image parameter; NS, not significant association with image parameter; ND, no association investigated

Another relevant nitroimidazole for hypoxia PET is 18F-fluoroazomycin-arabinoside (18F-FAZA), which is less lipophilic and may display better uptake kinetics compared to 18F-FMISO [58]. 18F-FAZA uptake has shown to be highly correlated with pimonidazole staining in, among others, head and neck and cervical cancer xenografts [58]. In a study on 15 patients with cervical cancer, 18F-FAZA PET was done before, during and after external chemoradiotherapy (Table 1) [59]. 18F-FAZA uptake was visually evaluated, and only five patients showed marked tumor uptake. These patients had somewhat larger tumor and higher proportion of failures compared to 18F-FAZA-negative patients, although the patient cohort was too small to make firm conclusions. Moreover, four out the five patients still had marked 18F-FAZA uptake into the course of fractionated radiotherapy, but all patients were PET-negative after treatment.

Vercellino et al. [60] employed 18F-fluoroerythronitroimidazole (18F-FETNIM) PET prior to chemoradiotherapy of cervical cancer (Table 1). Sixteen patients underwent hypoxia PET alongside 18F-FDG PET and analysis of osteopontin blood serum levels. Osteopontin was included as it has been associated with tumor hypoxia in head and neck cancer [61]. 18F-FETNIM tumor uptake was difficult to discriminate from adjacent normal soft tissues, indicating rather low tumor specificity. In comparison, all tumors showed pronounced 18F-FDG uptake. No correlation was seen between 18F-FETNIM uptake and 18F-FDG uptake or osteopontin level. However, patients with high 18F-FETNIM uptake had significantly reduced outcome, although the choice of threshold, i.e., tumor to muscle ratio (*T*/*M*), for assigning patients to the high-uptake risk group was not elucidated.

### Cu-ATSM as hypoxia tracer

**Fig. 1:**
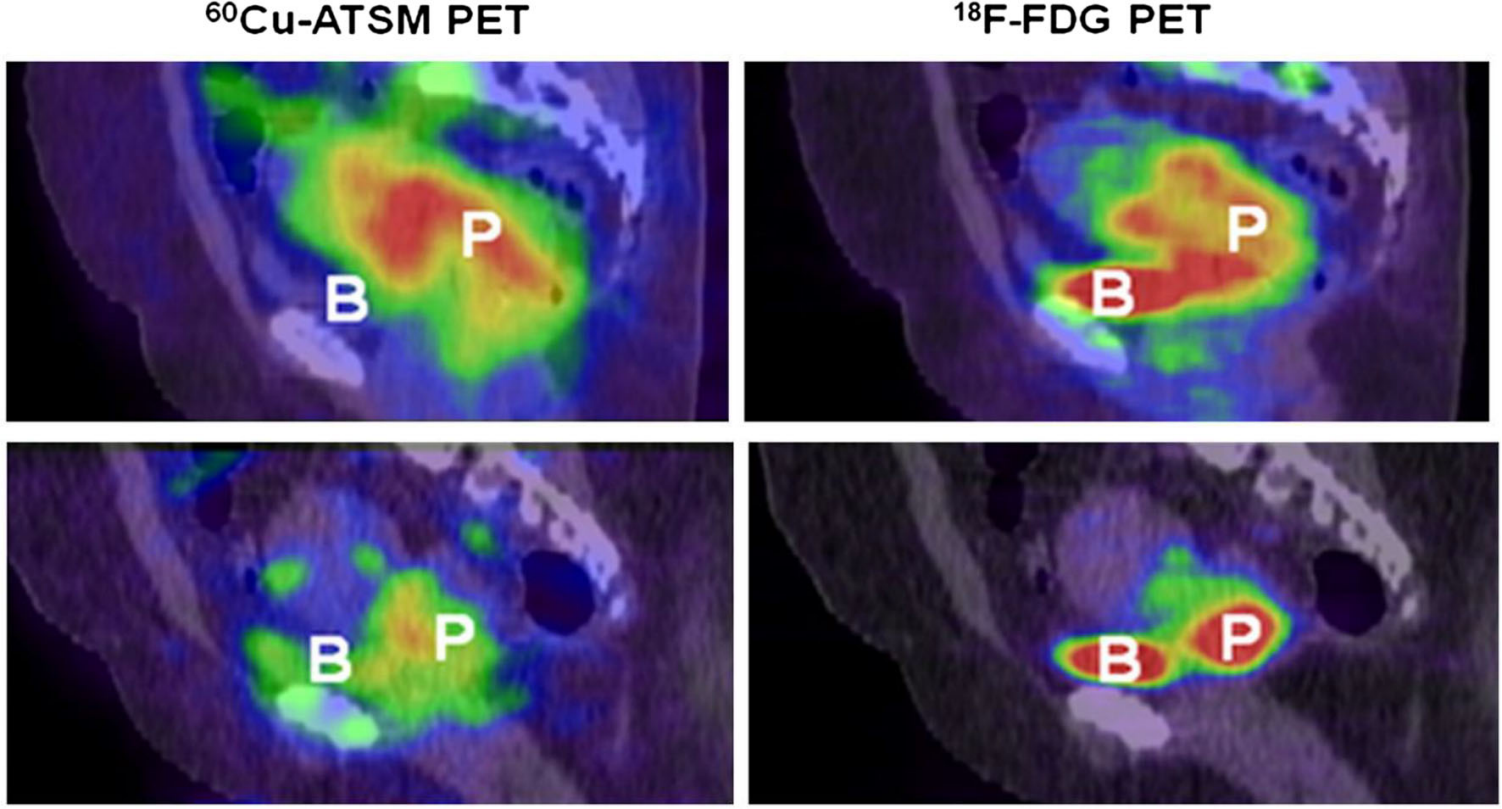
Comparison of 60CU-ATSM and 18F-FDG uptake in a hypoxic and normoxic cervix tumor. *Upper* hypoxic tumor. Sagittal 18F-FDG PET/CT image (*right*) of pelvis, showing high 18F-FDG uptake in tumor. Sagittal 60Cu-ATSM PET image coregistered with CT image (*left*) at same level, also showing high tumor uptake of this tracer (*T*/*M* = 4.5). *Lower* normoxic tumor. Sagittal 18F-FDG PET/CT image (*right*) of pelvis, showing high 18F-FDG uptake in tumor. Sagittal 60Cu-ATSM PET image coregistered with CT image (*left*) at same level, showing only mildly increased tumor uptake of this tracer (*T*/*M* = 3.0). Note that there are different patterns of 18F-FDG and 60Cu-ATSM uptake in both tumors. *P* tumor, *B* bladder. This research was originally published in *JNM* [62]. © by the Society of Nuclear Medicine and Molecular Imaging, Inc

The largest study conducted on hypoxia PET in cervical cancer to date is the multicenter trial on 60Cu-ATSM initiated at Washington University (Table 1) [53, 62–64]. In this trial, 60Cu-ATSM PET was conducted in dynamic mode, acquiring data from 0 to 60 or 30 to 60 min post injection. This rather short time interval between injection and imaging compared to nitroimidazole-based PET reflects the faster biokinetics of 60Cu-ATSM. Results from 38 patients were reported in the final publication [62]. The published studies have shown that virtually all tumors have significant Cu-ATSM uptake, with a mean *T*/*M* of nearly 4. Patients with high *T*/*M* had significantly lower progression free survival compared to those with low *T*/*M*. Moreover, there was no correlation between the 60Cu-ATSM data and glucose uptake assessed by 18F-FDG PET. This is exemplified in Fig. 1, showing PET images of two patients with glucose-avid tumor being non-hypoxic and hypoxic, respectively. In a subgroup analysis (*n* = 15) comparing biopsy-based immunohistochemical markers and 60Cu-ATSM PET, patients with more hypoxic tumors had significantly higher CA9 expression compared to those with less hypoxia [64].

Although the 60Cu-ATSM work is the most extensive study on hypoxia PET in cervical cancer, it is challenging to evaluate the potential of this tracer in future trials. Judging from pre-clinical (non-cervix) data, intratumoral uptake patterns of 18F-FMISO and 64Cu-ATSM are similar, with slightly higher 64Cu-ATSM uptake, but the concordance varies between xenograft models [65–67]. Furthermore, carbogen breathing, known to cause elevation of tumor oxygen levels, gave increased staining of a nitroimidazole-based immunomarker but no change in 64Cu-ATSM uptake [67]. In addition, Cu-ATSM uptake may be perfusion dependent [67], which was not the case for 18F-FMISO evaluated more than 2 h after tracer administration [52].

### Fluorodeoxyglucose (FDG) as a potential surrogate hypoxia tracer

Cervix tumors are generally glucose avid, showing high signal intensity on 18F-FDG PET [68, 69]. For the last two decades, there has been an ongoing discussion on the potential use of 18F-FDG as a marker of tumor hypoxia [70–73]. Normal cells will indeed turn to glucose metabolism for ATP production when exposed to hypoxia (Pasteur effect). However, tumor cells, even at normoxic conditions, very often utilize glucose metabolism for ATP synthesis (Warburg effect). Thus, any increase in 18F-FDG uptake due to hypoxia may well be obscured by that aerobic glycolysis already is activated. Moreover, common signatures of both hypoxia and aerobic glucose metabolism, such as GLUT1, HK2, and HIF1A expression, are often found to be elevated in cervix tumors [28, 74, 75]. Thus, depending on underlying activation of regulatory pathways, co-existence of hypoxia and aerobic glycolysis may be present in some tumors or tumor regions, but not in others. As virtually all cervix tumors are glucose avid, but not all are hypoxic, it is, therefore, not possible to pinpoint individual hypoxic tumors by 18F-FDG PET with high accuracy. This is further exemplified in the literature on hypoxia PET tracers (Table 1), reporting varying degree of correlation between uptake of hypoxia tracer and 18F-FDG.

## BOLD—blood oxygenation level-dependent MRI and related techniques

In MRI, the paramagnetic properties of mainly tissue hydrogen are probed using a combination of an external magnetic field, magnetic field gradients, and frequency- and phase-encoded radiofrequency (RF) waves. Following an RF pulse with resonance frequency, the hydrogen nuclei experience relaxation through two principally different modes: T1- and T2 relaxation or longitudinal and transverse relaxation, respectively. Relaxation rates are further influenced by other magnetic molecules in proximity, and MR image contrast arises from differences in hydrogen density and relaxation rates between tissues. In addition, inhomogeneities in the external magnetic field and local tissue magnetization will increase the transverse relaxation rate, leading to T2*-relaxation.

In blood vessels, hemoglobin (Hb) can be found as either oxyhemoglobin or deoxyhemoglobin (dHb), where oxygen-depleted tissues typically have high concentration of dHb. Deoxyhemoglobin is paramagnetic, and its presence will cause an increase in the T2*-relaxation rate R2* [76]. Thus, a decrease in blood oxygen saturation, indicating hypoxia, may be seen as a decrease in T2*-weighted image intensity or an increase in R2* image intensity. Therefore, sequences sensitive to blood saturation levels are denoted BOLD–blood oxygenation level-dependent MRI. BOLD MRI has shown significant association with tumor oxygen measures in both pre-clinical and clinical setting, although the literature is not fully consistent [76]. The feasibility of using BOLD in cervical cancer was investigated by Hallac et al. [77], where in total, nine patients were examined by two different MR acquisition techniques before and during oxygen breathing. Positive changes in BOLD signal intensity during breathing were found, albeit these changes were not highly tumor specific. A technique similar to BOLD, but which utilizes oxygen dependent T1-relaxation, has been denoted TOLD—tissue oxygen-level-dependent MRI. Here, increase in T1 relaxation is caused by the presence of dissolved oxygen [76]. The technique was employed on two patients with cervical cancer and eight patients with other abdominal cancers during oxygen breathing [78]. A significant increase in signal intensity was found for both cervix tumors and six out of the remaining eight tumors, illustrating sensitivity of the method to oxygen modification. Furthermore, some resemblance was found between the TOLD images and perfusion images derived from DCE-MRI.

The approach of using oxygen breathing during BOLD or TOLD image acquisition is cumbersome and not suited for clinical routine. Utilizing the relaxation rate directly as hypoxia measure would, therefore, be more optimal, but there are many unresolved issues regarding absolute quantification [76, 79]. Nevertheless, BOLD was used in 30 cervical cancer patients to assess changes in tumor oxygenation after radiotherapy (Table 1) [80]. First, tumor R2* was markedly higher after therapy, indicating increased dHb levels and thereby possibly more hypoxia. This apparently unexpected observation was later confirmed in a second study from the same institution [81], and the authors argued that treatment-induced vascular damage could be a contributing factor. Second, high R2* was negatively associated with the percentage of tumor shrinkage [80], in line with the hypothesis that hypoxic tumors (with high R2*) show poor response. This was further supported by a study on 65 patients [82], showing that non-responders had significantly higher R2* compared to responders. Moreover, in 30 patients with recurrent disease after surgery, hypoxic fraction derived from R2* mapping at the time of second line radiotherapy was significantly correlated with percentage of tumor shrinkage [83]. Thus, the body of literature on BOLD and related techniques in cervical cancer is more extensive and includes more patients than the literature on hypoxia PET tracers. Results from different studies are more or less consistent, warranting further clinical applications of this MR-based technique.

## Dynamic contrast-enhanced (DCE) imaging

For DCE imaging with either MR or CT, a bolus of contrast agent is administered intravenously into the patient and transported via the blood stream to the tumor [76]. The low molecular weight contrast agents used in clinical routine move freely across the vessel walls in tumors and distribute in the extracellular space before being gradually washed out. For MRI, paramagnetic contrast agents, typically based on gadolinium, cause a signal intensity increase in T1-weighted images. Conversely, the contrast in CT images is enhanced by iodinated contrast agents [84]. Rapid image acquisition is performed to track the contrast agent in tumor. Due to the highly permeable vessels in tumors, the uptake is perfusion limited, rather than limited by vessel permeability, and depends mainly on the blood flow, vascular density, and size of the extravascular, extracellular space [85]. For high molecular weight contrast agents, the tumor distribution is more dependent on vessel permeability. DCE imaging is unable to provide direct measures of tumor oxygenation. However, it is well documented that imaging with low molecular weight contrast agents can visualize tumor physiology related to hypoxia in cervical cancer.

### Image parameters and relationship to hypoxia

The easiest way of analyzing the transient uptake curves is by retrieving semi-quantitative measures such as the relative signal increase (RSI), the slope of the initial part of the curve, and the area under the curve over a certain time period (AUC). Such measures have been shown to correlate with *p*O_2_ or hypoxic fraction in both MRI [86, 87] and CT [88] studies on cervical cancer (Table 1). Hence, hypoxic tumors or tumor regions typically display low signal enhancement in DCE images. Moreover, RSI has been shown to be associated with cell density [87] and vessel density [89] in tumor biopsies, and, therefore, probably reflects both oxygen demand and supply.

**Fig. 2:**
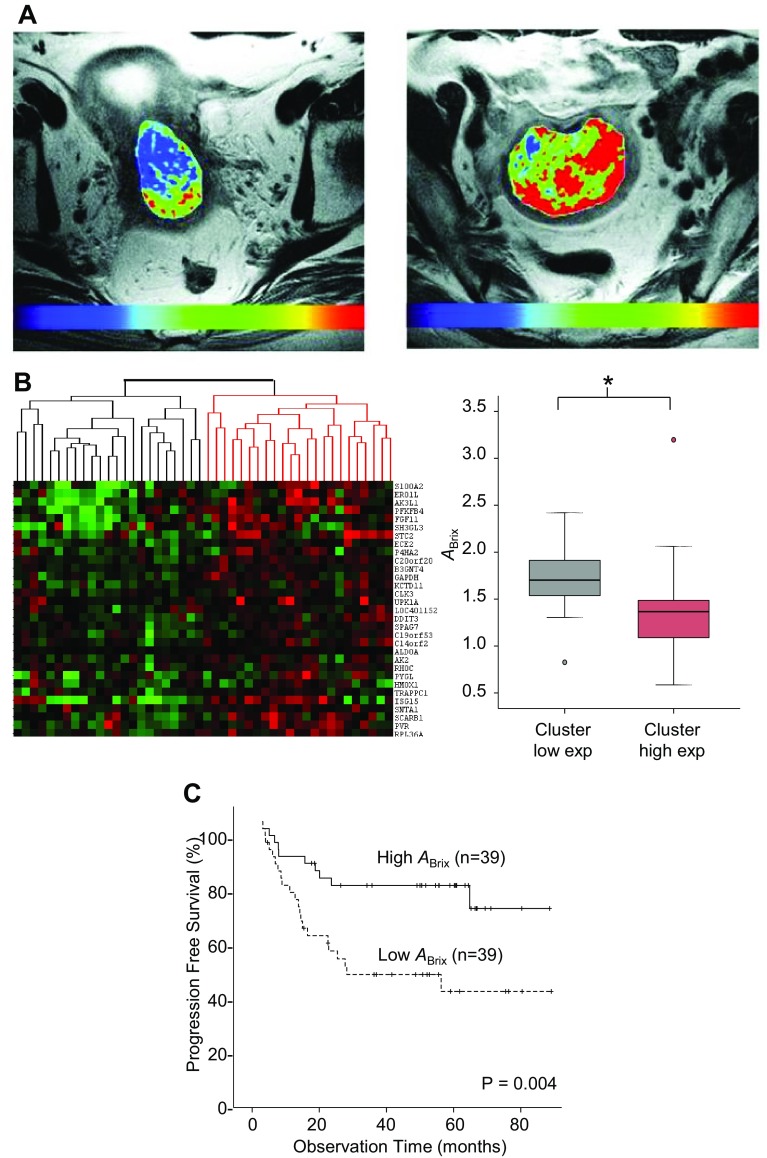
DCE-MRI parameter A_Brix_ in relationship to hypoxia gene expression and chemoradiotherapy outcome in cervical cancer. **a** Tumor A_Brix_ map superimposed on axial T2-weighted MR image of two different patients with more hypoxic (*left*) and less hypoxic (*right*) tumor. The *color scale* indicates A_Brix_ values in the range from 0 to ≥5.0. **b** Unsupervised clustering of 46 patients based on the expression of 31 hypoxia responsive, A_Brix_-associated genes (*left*). Box plot of A_Brix_ for the two patient groups identified by clustering, displaying lower A_Brix_ in cluster with high gene expression (*right*). **c** Kaplan–Meier curves for progression-free survival of 78 patients with low (below median) and high (above median) A_Brix_. *P* value from log-rank test and number of patients are indicated Reproduced with permission from Halle et al. [46]

Quantitative image parameters, derived by fitting the uptake curves to pharmacokinetic models, provide more specific physiologic information. The most used parameters, K^trans^ from the Tofts model and A_Brix_ from the Brix model [90, 91], reflect blood perfusion and a combination of perfusion and the size of the extravascular, extracellular space, respectively [85]. In a study on 78 cervical cancer patients, the two parameters were found to be significantly correlated (*r* = 0.56) [92]. In addition, the *ν*_e_ parameter from the Tofts model, which reflects the size of the extravascular, extracellular space, was found to be strongly associated with A_Brix_ (*r* = 0.68), but showed no relationship to K^trans^ [92]. Several studies have shown a relationship between K^trans^ or A_Brix_ and *p*O_2_ in cervix tumors (Table 1) [86–88]. The A_Brix_ relationship was further supported by work at our institution by combined analysis of DCE-MR images and biopsy derived gene expression profiles (Table 1; Fig. 2) [46]. A significant correlation between A_Brix_ and hypoxia gene sets was demonstrated, and tumors with low A_Brix_ had upregulation of hypoxia responsive genes, including HIF1 targets and UPR genes.

Although CT is a widely available modality, only few studies have explored the potential of DCE-CT to visualize hypoxia in cervix tumors [88]. A strong correlation between the DCE-CT and -MRI parameters has been found [93–95], and it seems that tumor hypoxia can be equally well determined from both modalities. The CT approach is easy to implement due to the linear relationship between attenuation numbers and tracer concentration. DCE-MRI, on the other hand, benefits from the lack of radiation exposure and easy integration with T2 and DW images acquired during the same examination.

### Relationship to outcome

A potential clinical relevance of the above findings is demonstrated by a large number of studies showing significant associations between DCE-MRI parameters and clinical outcome (Table 1). In general, low pre-treatment contrast enhancement [96–98], A_Brix_, K^trans^, or related pharmacokinetic parameter [86, 92, 98, 99] has been shown to correlate with poor locoregional control or survival, as illustrated for A_Brix_ in Fig. 2. Most studies include 50 patients or more. Moreover, the fact that similar image parameters are addressed in different patient cohorts and at different institutions, strengthens the conclusions. In addition, Li et al. [100] investigated the relationship of DCE-CT images to outcome in 93 patients, and found that responders had significantly higher values of blood perfusion parameters than non-responders (Table 1).

There are also indications that images acquired during radiotherapy may have prognostic value [101, 102]. In a study of 98 patients, Mayr et al. [101] found that those with persistently low signal enhancement in DCE-MR images acquired before the start of treatment and in the early and mid-phase of the external radiotherapy had the highest risk of treatment failure. In contrast, patients with improved enhancement during therapy, probably reflecting reoxygenation, had favorable outcome, regardless of pre-treatment enhancement. Combining images acquired before and during radiotherapy may, therefore, improve identification of high-risk patients as compared to pre-treatment images. Although promising results, there is, however, no consensus on how to best analyse DCE images to derive the most robust response parameter reflecting hypoxia. Moreover, standardization of the parameters across different MR machines is an unresolved challenge.

## Outlook

### Other hypoxia PET tracers

For non-cervical cancer, several other hypoxia PET tracers than those mentioned above have been applied clinically [103]. 18F-based nitroimidazoles such as HX4 ([18F]3-fluoro-2-(4-((2-nitro-1H-imidazol-1-yl)methyl)-1H-1,2,3-triazol-1-yl)propan-1-ol) and EF5 ([18F]2-(2-nitro-1H-imidazol-1-yl)-*N*-(2,2,3,3,3-pentafluoropropyl)-acetamide) are available, and both are currently included in clinical studies on patients with cervical cancer (NCT02233387, NCT00978874). However, previous studies provide no evidences that these tracers are superior to the most widely used nitroimidazole, 18F-FMISO [103]. Furthermore, 124-Iodine (124I) is a long-lived positron emitter with chemical properties similar to 18F, and may thus allow for imaging at later timepoints after intravenous injection [104]. This could be an advantage for nitroimidazoles, having slow biokinetics. However, e.g., 124I-iodoazomycin arabinoside (124I-IAZA) displayed no favorable characteristics compared to 18F-FAZA in a pre-clinical model [105]. Furthermore, 124I-iodoazomycin galactopyranoside (124I-IAZGP), a tentative hypoxia marker, showed no significant tumor uptake in patients with colorectal and head and neck cancer [106]. Currently, there seems to be little clinical drive to implement these iodine-based tracers for hypoxia imaging. Thus, there are no indications in the literature of a preferred hypoxia PET tracer or group of tracers that should be tested clinically for cervical cancer.

### Combined imaging of flow and metabolism

As noted for DCE imaging, blood flow- or perfusion-based methods may provide surrogate maps of tumor oxygenation. However, the gold standard for assessing blood flow is PET with 15-Oxygen (15O)-labeled water as tracer [107]. It has been argued that regional flow–metabolism mismatch reflects an aggressive tumor phenotype that has adapted to a hypoxic environment [108, 109]. Thus, combined 15O-H_2_O and 18F-FDG PET may potentially be used for identifying such tumors. This approach was tested on ten patients with cervical cancer, where substantial regional variations in tumor flow and metabolism were found [109]. However, due to the short half-life of 15O, this approach is not feasible for most cancer centers. Still, studies have shown that ‘first pass’ DCE images after administration of contrast agent reflect tumor blood flow [107, 110]. In addition, different dynamic imaging principles such as MRI, CT, and PET may provide similar estimates of flow-related tumor parameters, as long as proper image acquisition and quantification protocols are followed [93, 94, 111, 112]. Thus, to identify regional flow–metabolism mismatch, one may combine DCE-MRI or DCE-CT with 18F-FDG PET, or perform full dynamic 18F-FDG PET. Using the latter, the patient can be examined in a single scan without the use of additional contrast agents. Hybrid PET/MR or PET/CT scan, where MR or CT is performed as a dynamic scan with contrast agent, may also be attractive. As 18F-FDG is the most widely available PET tracer and DCE methods are straightforward to implement, multiparametric imaging of flow and metabolism should be attractive for further studies addressing tumor hypoxia and aggressiveness in cervical cancer.

### Intravoxel incoherent motion (IVIM) DW-MRI

DW-MRI measures the motion of water molecules in tissues. The apparent diffusion coefficient (ADC) of water can be calculated from images with different diffusion weightings [113]. Traditionally, this parameter has been determined by fitting a monoexponential decay curve to plots of signal intensity versus diffusion weighting in terms of ‘*b*’ value. Due to high diffusion coefficient in necrotic tissue, it has been suggested that ADC values can be used to assess hypoxia [113]. Several studies have indeed reported an association between high pre-treatment ADC and poor outcome in cervical cancer [114, 115]. However, the opposite relationship has also been found [116, 117], probably reflecting an aggressive tumor phenotype with low ADC caused by high cellularity. Moreover, ADC has been shown to correlate with cell density in histological sections [118] and 18F-FDG uptake in PET images [119], but failed to correlate with *p*O_2_ [120] in cervix tumors. A better measure of hypoxia may, however, be achieved by analyzing DW images by the intravoxel incoherent motion (IVIM) model. Here, a biexponential model is employed to separate the blood flow component at *b* values lower than 200 s/mm^2^, from the diffusion properties of the cellular matrix at *b* values higher than 300 s/mm^2^ [113]. The DWI-based perfusion fraction has been associated with perfusion parameters derived from DCE-MR images (RSI, AUC, A_Brix_, K^trans^) in cervical cancer [121, 122], and shown promise as early response indicator during chemoradiotherapy [123]. The IVIM technology, therefore, seems to have a potential in hypoxia imaging of cervix tumors that warrant further investigation.

### Combination therapy with hypoxia targeting drug

Combination therapy with radiation and hypoxia targeting drug is as a promising strategy to improve treatment outcome in cervical cancer, and a number of new drugs targeting hypoxia responsive pathways are currently being developed [124]. The optimal drugs for each patient may be decided based on genomic information in a tumor biopsy, which indicates resistance mechanisms at play in the hypoxic tumor. Combining biopsy data with imaging has been proposed as an essential part of a multifactorial decision support system in future radiotherapy planning, presumably leading to more informative decisions and precise selection of high-risk patients [125]. The approach has been evaluated based on a hypoxia gene classifier and DCE-MRI in a pilot study of 64 patients at our institution [45]. Most hypoxic tumors (93%) identified with imaging were detected by the gene classifier. However, the genes classified some additional tumors as hypoxic. The apparent misclassification seemed to be caused by the presence of small hypoxic subpopulations in the regions where the biopsies were taken [126], emphasizing the need for imaging to achieve information from the entire tumor.

**Fig. 3:**
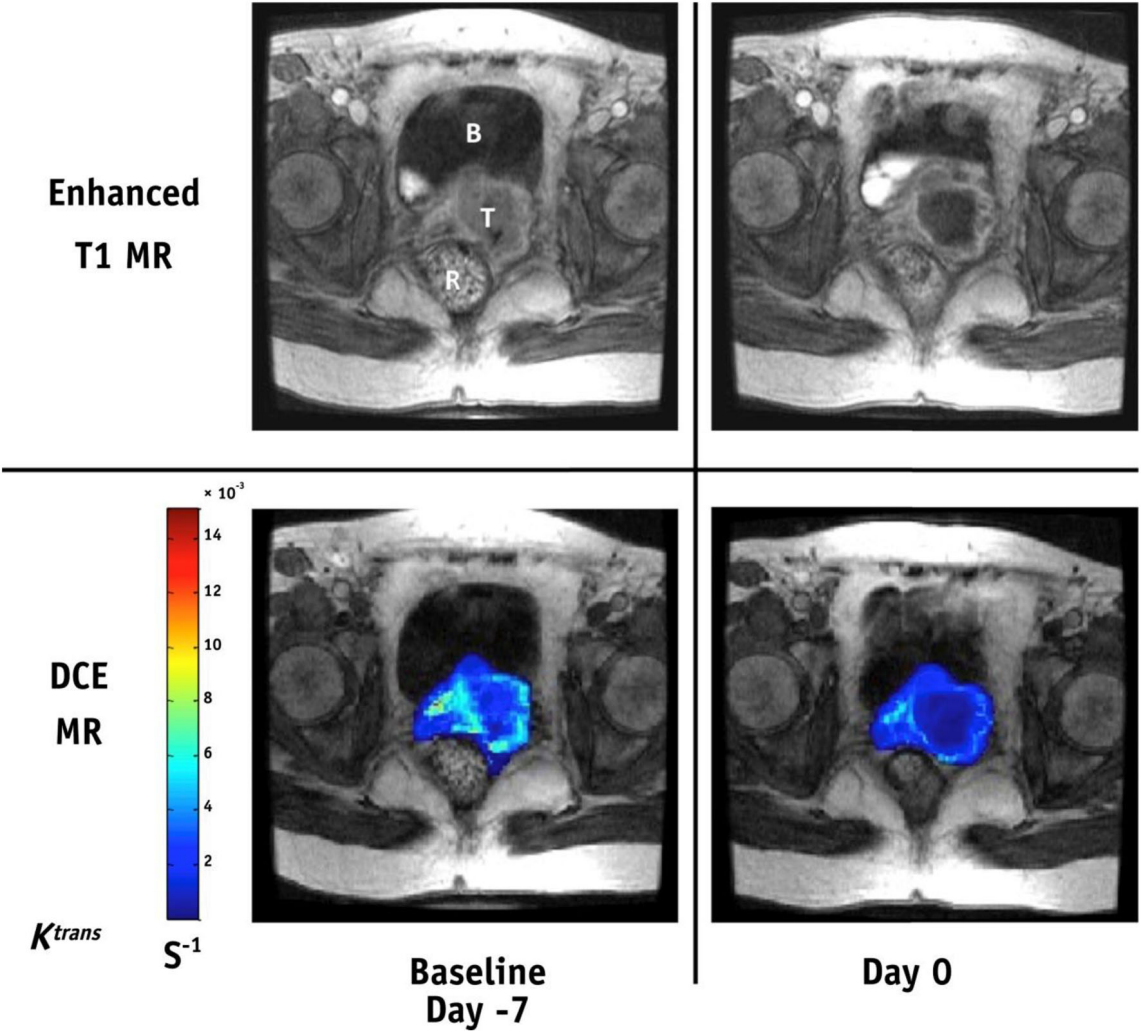
Changes in cervix tumor hypoxia by the angiogenesis inhibitor sorafenib, assessed by DCE-MRI parameter K^trans^. Late-phase axial DCE-MR T1 image of pelvis (*upper*) and the corresponding tumor K^trans^ maps (*lower*) superimposed on the late-phase DCE-MR T1 image at baseline before any treatment (day −7; *left*) and after 7 days of sorafenib alone (day 0; *right*). Note the decrease in K^trans^ after sorafenib treatment. *B* bladder, *R* rectum, *T* tumor Reproduced with permission from Milosevic et al. [8]

The potential of hypoxia imaging in the evaluation of drug effects in clinical trials was elegantly demonstrated in a recent study by Milosevic et al. [8]. They used K^trans^ parametric maps from DCE-MRI to record changes in hypoxia by the angiogenesis inhibitor sorafenib, as illustrated in Fig. 3. The drug was administered for 1 week prior to radiotherapy to 13 cervical cancer patients to tentatively reduce the amount of hypoxia in the tumors. The imaging data indicated that sorafenib alone increased, rather than decreased, the hypoxic fraction, as confirmed by parallel immunohistochemistry and oxygen electrode measurements. Sorafenib might, therefore, impair the outcome when combined with chemoradiotherapy. Based on this careful monitoring of the tumor hypoxia status, closure of the trial could be decided at an early timepoint.

### Radiotherapy dose painting

Radiotherapy dose escalation of resistant tumor regions, or dose painting, is an approach to increase the likelihood of tumor control without irradiating normal tissues to excessively high doses [127]. If hypoxic tumor regions are identified by imaging, the image information can be employed in digital radiotherapy planning systems to deliver escalated radiation doses locally to these regions by advanced radiotherapy techniques, such as volumetric modulated arc therapy or proton therapy [66]. Dose painting of hypoxic cervix tumors has been proposed [7], but has not been clinically implemented to date. This is mainly because local control rate after state-of-the-art combined chemotherapy, external radiotherapy, and brachytherapy is high and around 90% [128]. However, subgroups of patients with advanced and/or hypoxic disease have lower control levels [128], and patients with recurrent disease have few therapy options. By in silico radiotherapy planning, we have shown that external radiotherapy dose escalation of hyperglycolytic regions, identified by 18F-FDG PET, is feasible without increasing the dose to nearby organs [129]. This could lead to higher tumor control probability, with the highest expected gain for stage 3 and 4 tumors. In addition, it could also result in more extensive tumor shrinkage, facilitating less invasive brachytherapy. Although 18F-FDG PET is not a reliable measure of tumor hypoxia, our study shows the feasibility of dose painting in cervix tumors by external radiotherapy. Moreover, if tumor hypoxia is present at brachytherapy, i.e., after external radiotherapy, we have shown the feasibility to target DCE-MRI predicted hypoxic regions with elevated brachytherapy doses [130]. Thus, hypoxia dose painting may be attractive in future trials on cervical cancer, but more work is needed to identity high-risk candidates with persistent tumor hypoxia during treatment.

## Conclusion

Tumor hypoxia is a well-known risk factor for patients with cervical cancer, and much work has been done to develop hypoxia imaging procedures and strategies. PET with nitroimidazole tracers is regarded as the gold standard for non-invasive assessment of hypoxia, but the literature on cervical cancer is scarce, with small patient cohorts (*n* < 16), and is, therefore, inconclusive. The lack of PET studies may partly be explained by higher costs and limited availability of scanners and radiotracers. Still, for the few patients investigated with PET, 60Cu/64Cu-ATSM seems to be the most promising tracer candidate. Furthermore, as T2-weighted MRI has been the diagnostic imaging workhorse in cervical cancer for roughly a decade, implementation of MRI-based sequences, such as BOLD, DCE, and DW, for hypoxia-related studies has been facilitated. This is reflected in the larger body of evidence using surrogate MRI-based measures such as perfusion for hypoxia imaging. Although there is a lack of international protocols for standardized DCE-MR image acquisition and analysis, results point rather uniformly to the applicability of this modality. Still, contrast-induced nephrogenic toxicity can be a challenge in DCE-MRI. Methods employing endogenous contrast such as BOLD may, therefore, be preferable, although standardization issues prevail, and more work is needed before BOLD imaging is clinically mature. Furthermore, multimodal imaging of flow and metabolism may become attractive, as both hybrid PET/CT and PET/MR scanners can be used for this purpose. Imaging is expected to play a crucial role in future treatment strategies to target hypoxia in cervical cancer, such as drug-radiation combination therapies and radiotherapy dose painting approaches. The ultimate test to decide the optimal imaging method would be a randomized trial comparing hypoxia targeting to the standard treatment with multimodality imaging.
